# Temporal variations of δ^13^C-CH_4_ in rice paddies dominated by the plant-mediated pathway

**DOI:** 10.1016/j.isci.2025.112886

**Published:** 2025-06-16

**Authors:** Ji Li, Huilin Chen, Aijun Ding, Xuguang Chi, Weimin Ju, Yongguang Zhang, Philippe Ciais, Wenping Yuan, Shushi Peng, Zeqing Ma, Guirui Yu, Jing M. Chen

**Affiliations:** 1 Joint International Research Laboratory of Atmospheric and Earth System Sciences, School of Atmospheric Sciences, Nanjing University, Nanjing, China; 2 Frontiers Science Center for Critical Earth Material Cycling, Nanjing University, Nanjing 210023, China; 3 Jiangsu Provincial Collaborative Innovation Center of Climate Change, Nanjing, China; 4 Jiangsu Provincial Key Laboratory of Geographic Information Science and Technology, Key Laboratory for Land Satellite Remote Sensing Applications of Ministry of Natural Resources, School of Geography and Ocean Science, Nanjing University, Nanjing, Jiangsu, China; 5 Jiangsu Center for Collaborative Innovation in Geographical Information Resource Development and Application, International Institute for Earth System Sciences, Nanjing University, Nanjing, Jiangsu 210023, China; 6 Laboratoire des Sciences du Climat et de l’Environnement, LSCE/IPSL, CEA-CNRS-UVSQ, Université Paris-Saclay, Gif-sur-Yvette, France; 7 Climate and Atmosphere Research Center (CARE-C), The Cyprus Institute, Nicosia, Cyprus; 8 Institute of Carbon Neutrality, Sino-French Institute for Earth System Science, College of Urban and Environmental Sciences, Peking University, Beijing, China; 9 Laboratory for Earth Surface Processes, Peking University, Beijing, China; 10 Key Laboratory of Ecosystem Network Observation and Modeling, Institute of Geographic Sciences and Natural Resources Research, Chinese Academy of Sciences, Beijing, China; 11 College of Resources and Environment, University of Chinese Academy of Sciences, Beijing, China; 12 School of Geographical Science, Key Laboratory for Humid Subtropical Eco-Geographical Processes of the Ministry of Education, Fujian Normal University, Fuzhou 350008, China; 13 Department of Geography and Planning, University of Toronto, Toronto, ON M5S 3G3, Canada

**Keywords:** Isotope geochemistry, Isotope chemistry, Plant Biology, Biogeoscience, Biogeochemistry

## Abstract

The stable carbon isotope signature of methane (δ^13^C-CH_4_) helps constrain CH_4_ emissions on regional to global scales, yet observations in rice paddies remain limited. The temporal dynamics of δ^13^C-CH_4_ and its drivers in rice paddies are still not fully understood. Here, we conducted continuous *in situ* observations of CH_4_ and δ^13^C-CH_4_ at three canopy heights throughout a rice growing season. We investigated the emission source signature (δ^13^C_source_) and partitioned it into plant-mediated (δ^13^C_P_) and non-plant-mediated (δ^13^C_NP_) pathways. δ^13^C_source_ exhibited significant temporal and vertical variability, influenced by transport pathways and external sources. We found that temporal variations in δ^13^C_source_ were strongly correlated with rice photosynthesis and predominantly driven by δ^13^C_P_. Our results show that plant-mediated transport, rather than CH_4_ production, dominates δ^13^C_source_ variability. These findings provide new insight into the δ^13^C_source_ dynamics in rice paddies and will be crucial for improving regional-to-global estimates of CH_4_ fluxes from rice agriculture.

## Highlights

• Continuous *in situ* observations of CH_4_ and δ^13^C-CH_4_ were conducted in rice paddies
• Significant seasonal variations in δ^13^C_source_ are revealed
• δ^13^C_source_ is linked to rice photosynthesis and dominated by plant-mediated transport
• GPP is suggested as a potential proxy for CH_4_ emission at large spatial scales

## Graphical abstract

**Figure:**
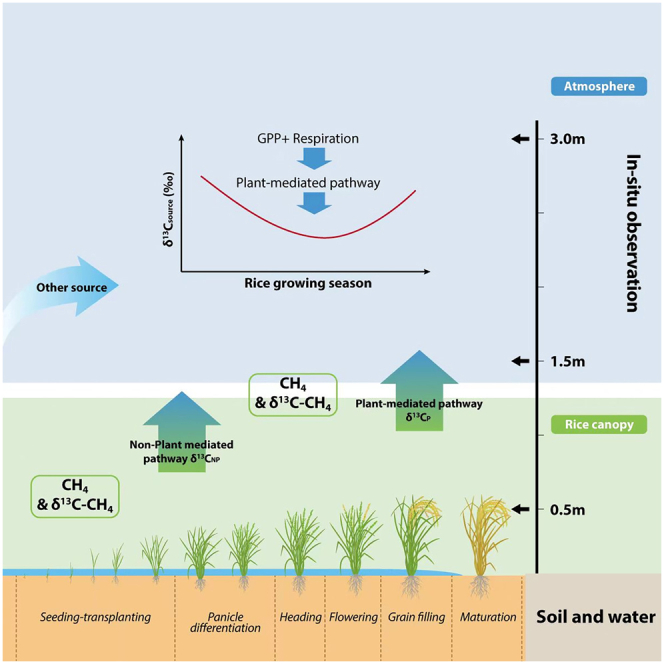

## Introduction

Methane (CH_4_) is an important greenhouse gas, accounting for ∼16.4% of radiative forcing.^1^ It has a global warming potential (GWP) of 30 and 83 times that of carbon dioxide (CO_2_) over 100-year and 20-year horizons, respectively.^2^^,^^3^^,^^4^ Therefore, reducing CH_4_ emissions is particularly crucial for achieving the Paris Climate Agreement Goals, especially for the near-term targets.^5^^,^^6^ Rice paddies are an important CH_4_ source, with an estimated emission rate of ∼30 [25–38] Tg CH_4_ yr^−1^ during 2008–2017,^7^ approximately 8% of global anthropogenic emissions. Accurately estimating the magnitude of CH_4_ emissions from rice paddies is crucial for assessing the global CH_4_ budget and evaluating regional emission reduction efforts. The δ^13^C signature of methane emissions from rice paddies (δ^13^C_source_) shows regional variation, typically ranging from −65‰ to −55‰. For example, studies have reported δ^13^Csource values of −63‰ to −58‰ in Asian rice fields,^8^^,^^9^ while slightly enriched values around −57‰ to −55‰ have been observed in European and North American rice paddies.^10^^,^^11^ These variations reflect differences in microbial pathways, rice varieties, and water management practices across regions. The estimation of CH_4_ emission intensity from rice paddies relies on bottom-up and top-down approaches. The bottom-up approach is based on either inventories^12^^,^^13^ or ecosystem process models.^14^^,^^15^^,^^16^ The top-down approach uses atmospheric transport and chemistry models to optimize CH_4_ fluxes based on atmospheric observations and prior emissions,^17^^,^^18^^,^^19^ which faces challenges in separating the emissions from various sources.^20^^,^^21^ The stable carbon isotope signature of CH_4_ emission source (δ^13^C-CH_4_, henceforth δ^13^C_source_), which is distinct among emission sources, is widely used as an independent constraint on CH_4_ emissions and sinks in global CH_4_ studies.^7^^,^^9^ Accurately determining δ^13^C_source_ is the key to accurately separating CH_4_ emissions from rice paddies on regional to global scales. However, the observations of rice paddy δ^13^C_source_ values are sparse, and constant global average values of ∼ −60‰ are used in recent global CH_4_ budget studies,^20^^,^^22^ without considering its temporal variation.

**Figure 1:**
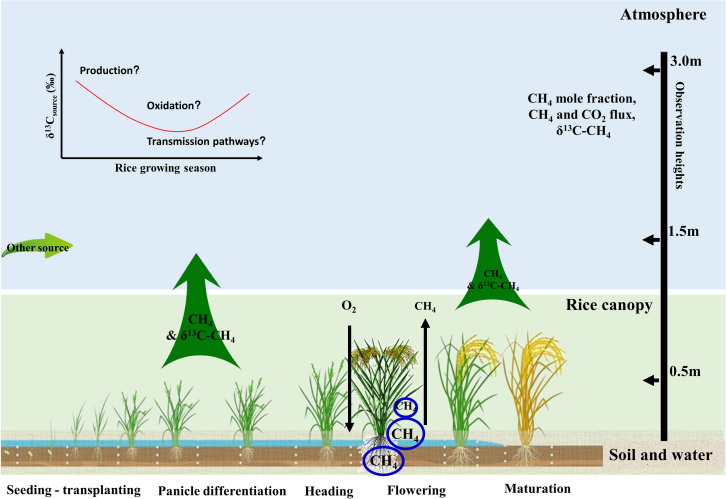
Temporally varying δ^13^C_source_ from rice paddies and possible influencing factors Field measurements of CH_4_ mole fraction and δ^13^C_obs_ were conducted at 0.5 m, 1.5 m, and 3.0 m, with CH_4_ and CO_2_ flux measurements at an eddy flux tower at 3.0 m. The δ^13^C_source_ for CH_4_ emitted from rice paddies was supposed to be temporally varying, which was influenced by CH_4_ production, oxidation, and three different transmission pathways that transported CH_4_ from the soil to the atmosphere.

On the rice field scale, δ^13^C_source_ can be affected by factors that are related to rice growth, including different CH_4_ production pathways, fractionation during CH_4_ oxidation, and different pathways through which CH_4_ is transmitted to the atmosphere.^23^^,^^24^^,^^25^^,^^26^^,^^27^^,^^28^^,^^29^ Therefore, δ^13^C_source_, providing crucial insights into the temporal dynamics of CH_4_ emissions from rice fields, is the results of the combined effects of isotopic fractionation in CH_4_ production, oxidation, and transmission, and may vary with the evolving stages of rice growth (Figure 1). However, how δ^13^C_source_ is regulated by CH_4_ production, oxidation, and transmission is still not fully understood.

CH_4_ emitted from rice paddies is transported through several pathways, including diffusion through diffusion, ebullition, and plant-mediated transport via rice aerenchyma. These transport mechanisms are associated with different levels of isotopic fractionation, leading to distinct δ^13^C source signatures. These transport mechanisms are associated with different degrees of isotopic fractionation, leading to distinct δ^13^C source signatures.^30^^,^^31^ Since the individual contributions of diffusion and ebullition are generally small during the rice growing season and are difficult to distinguish based on direct observations, they are collectively treated as the non-plant-mediated transport pathway in this study. Characterizing the isotopic signatures associated with different CH_4_ transport pathways is essential for interpreting the dynamics and drivers of methane emissions in rice paddies. The promotion effect of gross primary productivity (GPP) and the total ecosystem respiration on CH_4_ fluxes from rice paddies has been found.^32^^,^^33^^,^^34^^,^^35^ However, the relationship between δ^13^C_source_ and GPP or respiration is unclear. Understanding this relationship and the mechanisms behind it could offer new insights and methods for determining global and regional δ^13^C_source_ in rice fields or vegetated wetlands based on a comprehensive GPP database.

Here, we aim to investigate the temporal variation of δ^13^C_source_ and its influencing factors. We reveal temporal variations of CH_4_ and δ^13^C-CH_4_ observations above a rice paddy throughout the growing season in 2018. We derive the isotopic source signature of CH_4_ emitted through the plant-mediated pathway (δ^13^C_P_) and non-plant-mediated pathway (δ^13^C_NP_), and obtain the overall δ^13^C_source_. Then, we analyze the correlations of δ^13^C-CH_4_ with GPP and respiration, and discuss the effect of GPP and respiration on δ^13^C_source_.

## Results and discussion

### Temporal variations of CH_4_ and δ^13^C-CH_4_ observations

**Figure 2:**
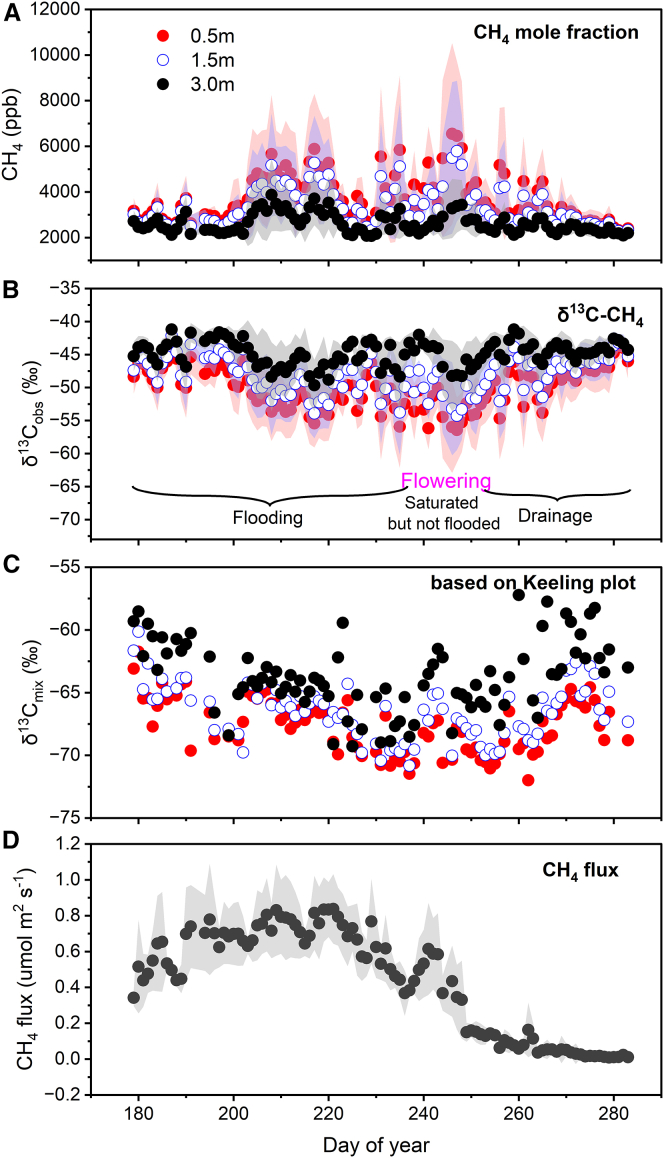
Measured daily CH_4_ mole fractions, δ^13^C-CH_4_, and CH_4_ fluxes during the entire rice growing season in 2018 The measurements of CH_4_ mole fraction, δ^13^C_obs_, and δ^13^C_mix_ at 0.5 m, 1.5 m, and 3.0 m are shown as circles in red solid, blue open, and black solid, respectively. δ^13^C_mix_ was derived from the Keeling plot approach using 5-min averages of CH_4_ mole fractions and δ^13^C-CH_4_ within each day, and with a threshold of R^2^ > 0.5. δ^13^C_mix_ on 0.5 m, 1.5 m, and 3.0 m are represented by solid red, open blue, and solid black circles, respectively. The daily CH_4_ flux is shown as a dark gray solid circle. The measurements starting from rice transplanting (DOY 175) were made by an isotopic cavity ring-down spectrometer and were averaged to obtain daily means. The uncertainties are represented as 1-sigma standard deviation in shaded areas.

The continuous atmospheric measurements of both CH_4_ mole fractions and δ^13^C_obs_, as well as ecosystem fluxes of both CO_2_ and CH_4_ at a rice paddy field in the Jurong Observation Station (31°48′24.59′N, 119°13′2.15′′E) show distinct seasonal variations (see Figures 2A and 2B). The CH_4_ mole fractions are enhanced between the tillering (around DOY 200) and the flowering period of rice (around DOY 250), while δ^13^C_obs_ is strongly anticorrelated with CH_4_ mole fraction (R^2^ = 0.85 at 0.5 m, R^2^ = 0.85 at 1.5 m, and R^2^ = 0.80 at 3.0 m (*p < 0.01*). The CH_4_ emission flux during the flooding period initially increases and then decreases over the growing season, and it continues to decline during drainage after the flowering stage (Figure 2D). Possible factors influencing the variations in both CH_4_ emissions and δ^13^C_obs_ include meteorological conditions, water management practices, substrate for methanogenesis, methanotrophy, and rice growth.^28^^,^^36^^,^^37^^,^^38^^,^^39^

The observed CH_4_ mole fraction enhancements and δ^13^C_obs_ depletion reflect shifts in the processes governing CH_4_ emissions at the temporal scale, including production, oxidation, and transmission.^40^^,^^41^^,^^42^ CH_4_ concentration reflects the magnitude of CH_4_ fluxes and mixing with surrounding air masses. Specifically, CH_4_ fluxes and wind speed determine the seasonal variation and short-term fluctuations of CH_4_ mole fractions, respectively. During the rice growing season, the observed CH_4_ mole fraction was positively correlated with CH_4_ fluxes, and negatively related with horizontal wind speed and friction velocity (u∗) across all three observation heights in both the beginning and the end of the growing season (Tables S1 and S3). However, in the middle of the growing season (DOY220 - DOY240), the correlation between CH_4_ mole fraction and horizontal wind speed increased (for example, from 0.38 to 0.55 at 1.5 m), while the correlation between CH_4_ mole fraction and CH_4_ flux weakened (Table S1). This change coincided with a notable fluctuation in horizontal wind speed and u∗ (Figure S1) and was also the reason for the fluctuation in CH_4_ mole fraction in the middle of the growing season.

Water management is a crucial factor controlling CH_4_ emission from rice paddies. Flooded rice field provides an anaerobic environment for CH_4_ production. The field in our study was continuously flooded before the flowering period, and drainage started afterward until the end of the growing season (see Figure 2). The soil water content (SWC) based on five depths decreased from the rice filing stage (Figure S1). SWC is not a limiting factor for CH_4_ production and transmission in the flowering period and before, with no significant correlations between SWC and CH_4_ emission flux. However, after the flowering period, as SWC decreased, CH_4_ emission flux decreased while δ^13^C_obs_ increased (Figure S1). We did not observe an increase in CH_4_ emissions shortly after drainage as expected by increased diffusivity when the soil got unsaturated, in contrast to the observations of Han et al.^36^

Rice growth is another major influencing factor through plant transport and substrate availability for methanogenesis. Numerous studies have demonstrated the impact of rice growth on CH_4_ emissions from rice fields.^24^^,^^43^^,^^44^^,^^45^^,^^46^ Rice growth influences CH_4_ emissions and their isotope composition in two ways. Firstly, it supplies through root exudates the carbon substrates for CH_4_ production.^43^^,^^47^^,^^48^ Secondly, it affects CH_4_ oxidation and transmission through the formation of rice aerenchyma.^27^^,^^29^^,^^44^ A positive correlation between GPP and CH_4_ flux was observed across the growing season, with R^2^ values ranging from 0.10 to 0.67 depending on the phenological stage (Table S1). The relationship was strongest during the early vegetative stage (R^2^ = 0.67, *p* < 0.01), indicating that photosynthetic activity significantly contributes to CH_4_ emissions, particularly during the early stages of rice growth. However, we are not able to quantitatively assess the relative importance of the GPP-induced changes of carbon substrates and the plant-mediated transport.

To obtain the stable carbon isotope source signature of CH_4_ from the rice paddy and conduct further quantitative analysis, we applied the Keeling plot method to 5-minute average measurements at each individual height (∼87 points) on a daily basis.^49^ The daily averaged δ^13^C_mix_, derived from Keeling plot method based on δ^13^C_obs_ and CH_4_ mole fraction (see Methods for equation, data resolution, and regression filtering criteria), exhibited a “U shape” seasonality for all three observation heights, ranging from −72.0‰ to −57.2‰ (see Figure 2C). The lowest δ^13^C_mix_ values were recorded around the rice heading period (from DOY 241 to DOY 250) (see Figure 2C). There are differences in δ^13^C_mix_ among the three observation heights, with heavier values at higher levels, and the differences increased after heading stage (Figures 2C, S2, and S12).

The differences in the δ^13^C_mix_ at different heights are surprising, as δ^13^C_mix_should reflect the isotopic compositions of CH_4_ mainly from the rice fields. The effect of observation height on the isotopic compositions of emitted methane has been observed in chamber studies.^36^^,^^50^ For wetlands, δ^13^C_mix_ values at 0.3 m and 3.0 m were −68.9‰ and −70.4‰, respectively.^50^ In a rice paddy, the values were more negative, −78.9‰ at 1.0 m and −77.4‰ at 5.0 m, respectively.^36^ These differences have been attributed to errors of observation or to uncertainty for the intercept of Keeling plots.^36^^,^^50^ As rice canopy height typically increases from 0.2 m to 1.1 m from transplanting to the filling period, selecting the observation height is crucial to accurately capture δ^13^C_mix_ from rice fields.

The differences shown in Figure S6 can be attributed to several factors. On one hand, measurements at different heights are influenced by different source areas, with sources possibly beyond the rice fields. On the other hand, isotope fractionation in different CH_4_ emission pathways contributes to the observed δ^13^C_mix_ differences. Understanding the contributions of CH_4_ emissions from plant-mediated and non-plant mediated pathways to δ^13^C_source_ is crucial and may be the key to explaining its temporal and vertical variations. Therefore, we further analyze δ^13^C_mix_ to separate the contributions of the two pathways.

In this study, CH_4_ emission sources were grouped into plant-mediated and non-plant-mediated pathways. Although the latter can include both diffusion and ebullition, we did not further separate them due to limitations of δ^13^C-based field observations, which cannot reliably distinguish between the two processes.^36^ In addition, this classification is consistent with the CH_4_MOD model structure, where ebullition is considered negligible and emissions are divided into two main pathways.^51^

### Variations and partitioning of δ^13^C-CH_4_ source signatures

We partitioned δ^13^C_mix_ as a mixture of 3 end-members, including CH_4_ sources of plant-mediated pathway (δ^13^C_P_) and non-plant-mediated pathway (δ^13^C_NP_), as well as minor contributions from other sources. We derived the following equations to describe the relationships among δ^13^C_P_, δ^13^C_NP_, and δ^13^C_mix_:

(Equation 1) δCmix13=δCP13×kp+δCNP13×knp+ε

(Equation 2) $$k_{p}+k_{\text{np}}=1$$

where $k_{p}$ and $k_{np}$ represent the fraction of CH_4_ emissions from plant-mediated and non-plant-mediated, respectively. The values of $k_{p}$ were simulated independently using two different process-based models: the IBIS (Integrated Biosphere Simulator) model^14^ and the BEPS-CH_4_ model.^52^ Both models incorporate a mechanistic representation of plant-mediated and non-plant-mediated CH_4_ transport processes and are driven by meteorological and remote sensing data.

The model-simulated $k_{p}$ values were then used in conjunction with our observations and Equations 1 and 2 to inversely estimate δ^13^C_P_and δ^13^C_NP_ every 15 days (one group) during rice growth season starting from the 3^rd^ day after rice transplanting. The possible range of δ^13^C_P_and δ^13^C_NP_ were set from −80‰∼−55‰ and −75‰ ∼−40‰, respectively.^28^^,^^53^^,^^54^ The parameter ε represents a residual correction factor accounting for small contributions from nearby non-paddy CH_4_ sources with distinct δ^13^C signatures, such as livestock operations, landfill sites, or fossil CH_4_ leaks.^10^^,^^11^^,^^55^ Although these sources were not dominant at the study site, their influence cannot be completely excluded. To ensure a conservative correction and minimize potential bias in δ^13^C_source_ estimation, ε was constrained to a range of 0‰–3‰. This range is supported by field observations from subarctic wetlands, where diel δ^13^C-CH_4_ variability of up to ∼4‰ has been reported due to intermittent non-local CH_4_ inputs and atmospheric mixing.^38^

**Figure 3:**
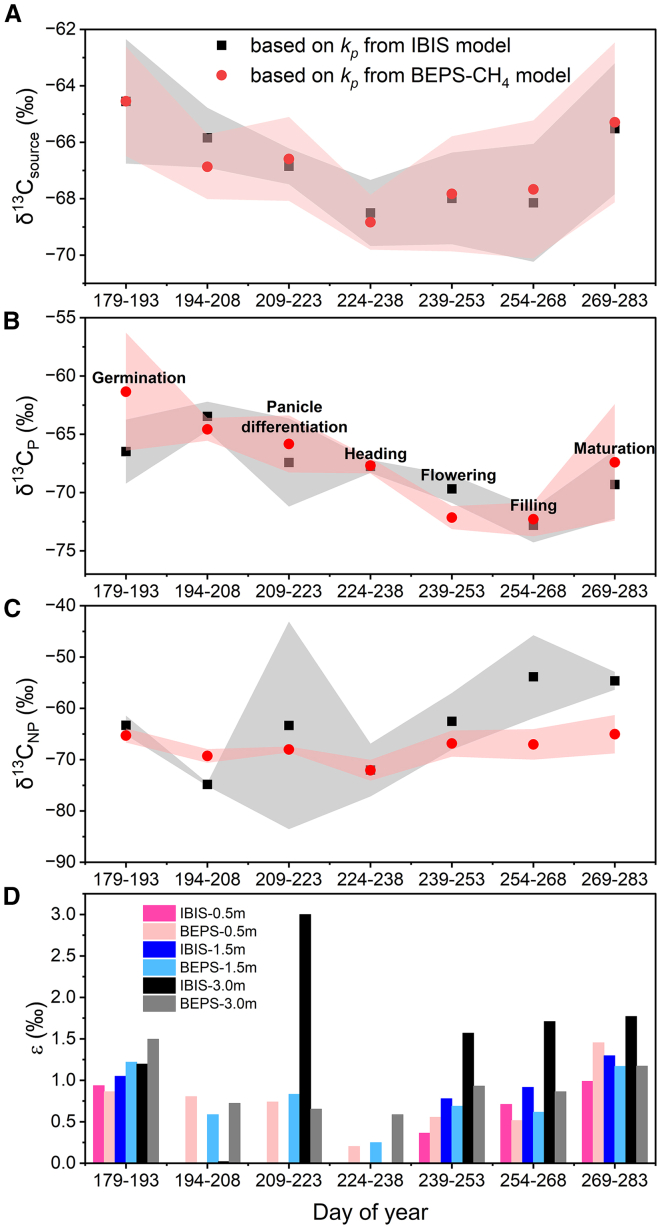
Seasonal variation of δ^13^C-CH_4_ source signatures from different emission pathways estimated by a mass balance method Seasonality of daily δ^13^C-CH_4_ source signatures by (A) weighted average method, with derived δ^13^C-CH_4_ source signatures for CH_4_ emitted through (B) plant-mediated and (C) non-plant mediated pathway, as well as (D) from other sources. According to the mass balance equation, δ^13^C_mix_ was divided into three main components, including δ^13^C_P_, δ^13^C_NP_, and ϵ, representing the δ^13^C-CH_4_ of CH_4_ emitted by plant-mediated pathway and non-plant mediated pathway, and other sources. The derivation details are described in “Methods.” *k*_*p*_ and *k*_*np*_ were calculated from the simulated CH_4_ fluxes through plant-mediated and non-plant-mediated pathways by the BPES-CH_4_ (the red circle) and the IBIS model (the black square). The weighted average δ^13^C value was calculated as the k_p_ and k_np_ weighted average of δ^13^C_P_ and δ^13^C_NP_, and was further averaged over 15 days. Data points shown in the Figure are the average of the simulations at three observation heights, and the uncertainties are represented as 1-sigma standard deviation in shaded areas.

The seasonal variation of weighted average δ^13^C_source_ (calculated as δCsource13=δCP13×kp+δCNP13×(1−kp)) ranged from −68.5 ± 0.97‰ to −64.5 ± 1.93‰, with the seasonal turning point at the heading stage (see Figure 3A). The δ^13^C_source_ values were more negative than the result directly derived from the observations using the Keeling plot method (δ^13^C_mix_), with a difference ranging from −1.3‰ to −0.3‰.

We found that δ^13^C_P_ decreased from the beginning of the growing season until the filling period (around 83 days after rice transplanting, or DOY 260) from −61.3‰ to −72.3‰, and bounced back in the rice maturity stage. The δ^13^C_P_ values were consistent when using the simulated fractions of plant-mediated emissions (k_p_) based on either the BEPS-CH_4_ or the IBIS model (Figure 3B). A few studies investigated the isotopic signatures of CH_4_ emitted from plant-mediated pathways.^24^^,^^28^^,^^56^ However, these studies typically involved sampling CH_4_ emitted from different parts of rice plants, soil, or field water, without continuous sampling throughout the entire rice growing season. The plant-mediated transmission relies on the formation and function of rice aerenchyma and is closely related to rice growth, reflecting seasonal variations.^23^^,^^27^^,^^29^

In contrast, δ^13^C_NP_ exhibits little seasonal variations. During the panicle differentiation stage, δ^13^C_NP_ based on k_p_ from the IBIS model showed relatively large uncertainty because the k_p_ during this stage was nearly a constant value within 33–47 days after rice transplanting (DOY 209–223) (Figures 3C and S3). Additionally, the impact of other sources during this period was relatively large (Figure 3D). The δ^13^C_NP_, including both ebullition and diffusion, was shown to have no obvious seasonality.^57^^,^^58^^,^^59^

We found that the enhanced fractionation of the plant-mediated pathway explains the observed decreasing trend of the δ^13^C_source_ until heading, and that the increasing oxidation and the weakening fractionation of the plant-mediated pathway determine the slight increase of the δ^13^C_source_ toward the end of the growing season. The source signature of δ^13^C_source_ from rice paddies is determined by fractionation during CH_4_ production, oxidation, and transmission processes. Firstly, two main types of methanogenic pathways in rice paddie soil are acetate- and H_2_/CO_2_-dependent methanogenesis, which exhibit different carbon isotope fractionation effects, with acetate-dependent methanogenesis being less negative.^60^^,^^61^ At the beginning of the rice growing season, H_2_/CO_2_-dependent methanogenesis plays a more important role, while acetate-dependent methanogenesis increases as rice growth and with continuous flooding, leading to an increasing role of acetate-dependent methanogenesis relative to H_2_/CO_2_-dependent methanogenesis.^29^ Therefore, the produced δ^13^C-CH_4_ will be less negative, i.e., increasing with rice growth. Secondly, the oxidation of CH_4_ in the soil leads to an increased δ^13^C value.^56^ According to the IBIS model results, the oxidation rate increases with rice growth until flowering and decreases toward the end of the growing season; the ratio of oxidation to production increases until panicle differentiation and then saturates during the rest of the growing season (Figure S4). Therefore, the oxidation process will result in an increase of δ^13^C-CH_4_ until flowering and a decrease of δ^13^C-CH_4_ afterward. Finally, the fractionation of the plant-mediated pathway increases until heading, e.g., from −14.7‰ to −16.7‰, and then decreased throughout the rest of the growing season,^28^^,^^56^ which results in a decreasing trend in the δ^13^C-CH_4_ of CH_4_ emitted through plant-mediated pathway.

Although diffusion and ebullition are distinct transport processes (e.g., ebullition tends to increase during the mid-to late-growth stages), we grouped them together as a non-plant-mediated pathway in our isotopic analysis. This choice reflects the limitation of δ^13^C-based field observations, which cannot isolate the isotopic signals of these individual components. While the IBIS model can separately simulate diffusion and ebullition, we adopted a simplified classification to align with the capability of our observational data. We recognize that this may underestimate the temporal variability of CH_4_ isotopic signatures, particularly during periods when ebullition is important. Future studies incorporating higher-frequency isotopic and flux measurements are encouraged to disentangle these processes.

### Correlations of δ^13^C-CH_4_ source signatures with gross primary productivity and TER

**Figure 4:**
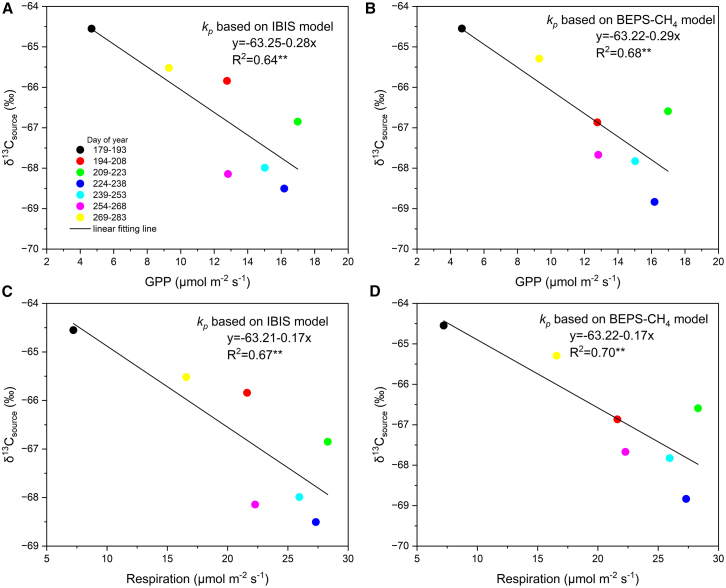
Correlations between GPP, Respiration, and δ^13^C_source_ in rice paddies δ^13^C_source_ was calculated from δ^13^C_P_ and δ^13^C_NP_, which were derived based on *k*_*p*_, from the IBIS-model (a, c) and (b, d) the BEPS-CH_4_ model, respectively. The different colors represent the data of a 15-day group. The uncertainties are reported as 1-sigma standard deviation.∗∗ indicates *p* < 0.01.

δ^13^C_source_ was negatively correlated with GPP and respiration during the entire rice growing season (Figure 4). During the mid-developmental period, δ^13^C_source_ reached its seasonal minimum, while both GPP and respiration peaked at their seasonal maxima. The significant correlation between LAI and δ^13^C_P_ was observed during the whole growing season (Figure S5). A reasonable assumption is that GPP affects δ^13^C_source_ in the following two ways. One is through the carbon fixed by leaf photosynthesis, which enters the soil as root exudates, increasing the substrate for CH_4_ production and altering the composition of organic carbon in the soil.^62^^,^^63^ Another way is to promote the formation and development of rice aerenchyma in the vegetative growth stage, thereby promoting plant-mediated CH_4_ transmission.^23^^,^^27^^,^^45^ While respiration competes with CH_4_ production in paddy soil for carbon sources.^64^

The observed relationships between GPP, Re, and methane emissions indicate that biotic processes play an important role in regulating CH_4_ fluxes from rice paddies. As an indicator of ecosystem productivity, GPP reflects the supply of photosynthates to the rhizosphere, thereby enhancing microbial activity and CH_4_ production. Re integrates both root and microbial respiration, representing carbon turnover that sustains methanogenesis. These findings highlight the importance of incorporating GPP and Re when interpreting methane emission dynamics, and suggest GPP’s potential as a proxy for estimating methane emission intensity at large spatial scales. We note that future studies could further explore the role of methanogenic substrates and plant-mediated mechanisms in shaping CH_4_ emissions.

The GPP-related formation and development of rice aerenchyma control the seasonal variations of both CH_4_ and δ^13^C_source_, although accurate partitioning of the impacts of GPP on the transport and the production of CH_4_ in rice paddies is challenging, which requires at least additional constraints on the temporal change of δ^13^C-CH_4_ in the soil. By comparing the seasonal variation of GPP and CH_4_ fluxes (Figures S1 and S6), it was found that GPP was not synchronized with CH_4_ production (Figures S1 and S6), but was rather corresponding with CH_4_ emission through plant-mediated pathway (Figure S13).

The rationale for using GPP to simulate CH_4_ source signatures lies in its dual influence on both methane production and transport processes. Higher GPP increases the allocation of recent photosynthates to roots, promoting root exudation and thereby enhancing the availability of labile carbon substrates for methanogenesis.^65^^,^^66^^,^^67^ Simultaneously, GPP is often correlated with the structural development of rice plants, such as aerenchyma formation, which facilitates plant-mediated CH_4_ transport.^24^^,^^46^^,^^68^ These two mechanisms together suggest that GPP not only reflects the potential for CH_4_ production but also modulates its emission pathways. Therefore, GPP-derived estimates of δ^13^C_source_ could better capture the temporal and spatial dynamics of CH_4_ emissions, especially when integrated with isotopic or pathway-partitioning approaches.

The δ^13^C_source_ values for rice paddies used in recent global CH_4_ budgets were simply averages of reported constant values in previous studies, without considering their temporal variations.^7^^,^^9^^,^^11^^,^^20^^,^^22^^,^^69^ However, seasonal variations in δ^13^C_source_ of rice paddies may be important in global and regional models. Furthermore, Asian countries account for more than 90% of the world’s total rice production^70^^,^^71^ and 30%–50% of global CH_4_ emissions.^7^ Based on the reported δ^13^C_source_ of major rice-producing countries (Table S2), we obtained the country-average δ^13^C_source_ and annual CH_4_ flux (Figure S7), and found that the δ^13^C_source_ and annual CH_4_ flux differed among these five Asian countries, ranging from −54 to −62.4‰ (Figure S7). For China, the significant difference in δ^13^C_source_ existed in South China (representative area: Hong Kong, −58.2‰) and East China (representative area: Jiangsu Province, −65.6‰); however, the global and Asian flux-weighted δ^13^C_source_ throughout the rice growing seasons were almost the same (Figure S8). Relying solely on global-average δ^13^C_source_ can lead to the loss of unique information across regions and seasons, and thus, we recommend using GPP models to simulate the ^13^C source signatures of CH_4_ from rice paddies.

### Conclusions

Overall, we present our findings on the significant temporal variation of the CH_4_ and δ^13^C_source_ throughout a growing season and its strong correlation with gross primary productivity (GPP). We found that the plant-mediated pathway component (δ^13^C_P_), rather than CH_4_ production, predominantly influences δ^13^C_source_. Our results offer a novel approach to understanding δ^13^C_source_ in rice paddies, which is crucial for accurately constraining CH_4_ fluxes on regional to global scales.

### Limitations of the study

This study was conducted at a single observation site, which may limit the representativeness of the results across varying soil textures known to influence CH_4_ production. Future studies across rice paddies with different soil types and environmental conditions are needed to improve the generalizability of δ^13^C_source_ dynamics. Due to observational constraints, ebullition and diffusion pathways were grouped as non-plant-mediated transport in this study. Given their distinct isotopic signatures, separating them through targeted measurements could clarify their individual contributions and associated isotopic fractionation. In addition, while a strong correlation between δ^13^C_source_ and rice photosynthesis was observed, the underlying mechanisms remain uncertain. Photosynthesis may influence δ^13^C_source_ either through changes in plant structure (affecting gas transport) or via root-derived carbon input to methanogenic substrates. Elucidating these processes through physiological and isotopic experiments would enhance our understanding of the role of GPP in CH_4_ emissions.

## Resource availability

### Lead contact

Further information and requests can direct to Dr. Huilin Chen (huilin.chen@nju.edu.cn).

### Materials availability

This study did not generate new physical materials.

### Data and code availability

• All field observation data, including CH4 mole fractions, δ13C-CH4, CH4 fluxes, and meteorological parameters, have been provided in the supplemental Excel file (Data S2).
• Python scripts to analyze the data are provided. Data plotting was performed using the Origin software.
• The IBIS model with the CH4 module used in this study is based on previously published work.^14^ The BEPS-CH4 module was developed and applied in a Ph.D. dissertation.^52^ Both IBIS and BEPS simulation results have been included in the supplemental data.

## Acknowledgments

The study was supported by the 10.13039/501100012166National Key Research and Development Program of China (grant number 2022YFE0209100), the 10.13039/501100001809National Natural Science Foundation of China (grant numbers 13001179, 42475115, and U24A20590), the Fundamental Research Funds for the Central Universities - Cemac “GeoX” Interdisciplinary Program (grant numbers 14380205 and 2024300245), the Fundamental Research Funds for the 10.13039/501100012429Central Universities (grant number 14380235) and the project of Youth Crossdisciplinary Team of the 10.13039/501100002367Chinese Academy of Sciences of China (grant number 2023000126). This research was also supported by the Collaborative Innovation Center of Climate Change, Jiangsu Province.

## Author contributions

J.L. and H.C. conceived the study. J.L. conducted the field observations and performed the data analysis. J.L. and H.C. wrote the article. A.D., X.C., W.J., and Y.Z. contributed to the field experiment setup and data collection. W.Y. and W.J. provided model-based support for data interpretation. P.C., S.P., Z.M., G.Y., and J.M.C. contributed to article editing and scientific discussion. All authors reviewed and approved the final article.

## Declaration of interests

The authors declare no competing interest.

## STAR★Methods

### Key resources table

**Table:** 

REAGENT or RESOURCE	SOURCE	IDENTIFIER
**Deposited data**		

Field measurements of CH_4_ mole fractions, carbon isotope, fluxes and micrometeorological variables in rice paddy	This paper	Data S2
Global methane emissions from rice cultivation regions	EDGARv6.0	https://edgar.jrc.ec.europa.eu/
Summary of δ^13^C source values in global rice regions	This paper	See Table S2
k_p_ values simulated using IBIS and BEPS-CH_4_ models	This paper	Data S2

**Software and algorithms**		

Cavity Ring-Down Spectroscopy Analyzer Software (for Picarro G2201-i)	Picarro Inc.	Included with instrument
Valve sequencer control software (sampling control)	Picarro Inc.	Included with instrument
MATLAB R2022b (data processing)	Commercially Available Software (Mathworks)	https://www.mathworks.com/
Python 3.9 (statistical analysis and scripting)	Python Software Foundation	https://www.python.org
OriginPro 2021 (data visualization)	OriginLab Corporation	https://www.originlab.com
IBIS model with CH_4_ module	Song et al., 2020^14^	https://doi.org/10.1029/2019MS001867
BEPS-CH_4_ module	Dai, 2019^52^	Archived at Nanjing University
fit_delta13C_components.py (nonlinear mixing model for δ^13^C components)	This paper	Included in Data S1
keeling_plot_fit.py (Keeling plot source signature estimation)	This paper	Included in Data S1

### Method details

#### Field measurements for CH_4_ mole fractions and carbon isotope

Field measurements on CH_4_ mole fractions and its stable isotopic composition of carbon (δ^13^C-CH_4_, henceforth δ^13^C) were performed in an irrigated rice (*Oryza sativa*) paddy field at the Jurong Observation Station (31°48′24.59′N, 119°13′2.15ʺE). The *in situ* observations began with the transplantation of rice on Day of Year (DOY) 176 in 2018 and continued until the rice reached full maturity on DOY 283 in 2018. The growth of rice was categorized into three main stages (Table S4).^65^ A wavelength-scanned cavity ring-down spectrometer (CRDS, Picarro, Inc., CA, USA, model G2201-i) was used to measure the vertical profiles of CH_4_ mole fractions and its corresponding δ^13^C-CH_4_ in the rice paddy field. This setup included a six-port manifold made of a series of solenoid valves, which allowed for sequential sampling of ambient air at three heights (0.5 m, 1.5 m, 3.0 m) and of three reference gases with distinct CH_4_ mole fractions (as detailed in Tables S5 and S6). Humid air samples were delivered directly to the CRDS for measurements (Figures S9A–S9D). The switching of the sampling lines was achieved by the valve sequencer software in the G2201-i analyzer. Air samples from each height were measured for 5 min. After every 600 min of ambient air sampling, the analyzer was calibrated with the three reference gases, each for 10 min. The CH_4_ concentrations of the three reference gases were 2004.32 ppb, 3592.80 ppb, 5017.03 ppb, respectively, and were calibrated using the same G2201-i spectrometer analyzer in laboratory (as illustrated in Figure S9E).

#### Data processing

For each observation height, the measurements were calculated by averaging the data from the last 2 min of each 5-min observation interval. Similarly, for the reference gases, we averaged the data from the last 5 min within each 10-min interval for further analysis. The resulting datasets were then corrected for water vapor effects and instrument drifts to obtain a time series of 5-min averages for each individual measurement height.

#### Water vapor correction

To derive the dry mole fractions of both ^12^CH_4_ and ^13^CH_4_, we followed the experimental methodology outlined by Chen et al. (2010) and Rella et al., (2013). Air from a cylinder was humidified by deionized water absorbed by cotton wool in the inlet tubing before analysis by a Picarro-G2201i analyzer in the laboratory. Throughout the experiment, water vapor was continuously added into the air stream, resulting in slowly decreasing water vapor levels as the cotton wool progressively dried out. The water vapor level varied from 3% to 0.02% during our experiment. The quadratic empirical formulas were built to obtain dry ^12^CH_4_ and ^13^CH_4_.^66^

To correct for the interference of water vapor on the measurements of ^12^CH_4_ and ^13^CH_4_ dry mole fractions using the Picarro G2201-i analyzer, we conducted a 24-h laboratory calibration experiment using a single standard gas, during which the water vapor concentration was gradually reduced from ∼3% to ∼0.02%. Based on these measurements, we derived empirical ratio-based correction formulas to convert wet mole fractions to dry mole fractions of CH_4_ isotopologues, using the analyzer-reported water vapor concentration (H_2_O) as the independent variable:

(Equation 3) C12H4wetC12H4dry=1.0000−0.0099·H2O−0.0003·H2O2

(Equation 4) C13H4wetC13H4dry=1.0000−5.0344×10−5·H2O−0.0003·H2O2

These corrections were applied to 5-min averaged data. H_2_O was taken directly from the raw output of the analyzer and used because it is the only humidity variable available in our field dataset.

#### Instrument drift correction

After the water vapor correction, we used the measurements of the three reference gases every 300 min to correct instrument drifts. The measurements of each reference gas were first interpolated to the entire measurement period, and then a linear relationship between the calibrated values and the measured or interpolated values of the reference gases was established for each 5-min measurement step. The linear calibration equations were used to drift-correct and calibrate the air sample measurements. CH_4_ mole fractions were reported on the World Meteorological Organization (WMO) X2004A scale. Carbon isotopic compositions (δ^13^C) were reported relative to the VPDB scale, calibrated against working standards that are traceable to the INSTAAR laboratory scale.

#### Estimation of δ^13^C_mix_ using the Keeling plot approach

Using the calibrated CH_4_ mole fraction of ambient air measurements of ^12^CH_4_ and ^13^CH_4_, we calculated δ^13^C of the observed CH_4_ (henceforth δ^13^C_obs_) at the three heights according to Equation 5.

(Equation 5) δCobs13=CH4cal13/CH4cal12(CH413/CH412)ref−1

Here, ‘ref’ denotes the ^13^C/^12^C ratio of the VPDB reference scale. The superscript ‘cal’ denotes the calibrated and water-corrected mole fractions of ^12^CH_4_ and ^13^CH_4_, after applying both water vapor correction (Equations 4 and 5) and standard gas calibration.

Based on this, the mixing carbon source signature (δ^13^C_mix_) of the rice field CH_4_ emissions is calculated using the Keeling plot method with the time series of measurements at individual heights,^45^^,^^46^^,^^67^ as follow,

(Equation 6) δCobs13=CH4bgCH4obs(δCbg13−δCmix13)+δCmix13

where, ${\text{CH}4}_{\text{bg}}$ and ${\text{CH}4}_{\text{obs}}$ represent the background and the observed CH_4_, respectively. We derived daily δ^13^C_mix_ based on 5-min averaged observations of CH_4_ and δ^13^C_obs_ and used a threshold of R^2^ > 0.5, accounting for 88.68% of all fitting results, to filter the data (Figures S10 and S11).

#### Simulation of plant-mediated CH_4_ emissions

The process for simulating the isotopic signatures of CH_4_ emissions via plant-mediated and non-plant-mediated pathways based on δ^13^C_mix_ is described in Equations 1 and 2 in the main text. The values of *k*_*p*_ used in the isotopic mixing model were simulated independently using both the IBIS and BEPS-CH_4_ models. These two process-based models incorporate mechanistic representations of plant-mediated transport and diffusion/ebullition pathways, and are driven by meteorological and remote sensing inputs.

#### Flux and micrometeorological measurements

CO_2_ and CH_4_ fluxes obtained during the same period with CH_4_ mole fraction in the same rice paddy were observed with an eddy flux system from a 3.5 m tower, which consisted of (1) an ultrasonic anemometer (WindMaster Pro, Gill Instruments Limited, Hampshire, UK), for three-dimensional wind speed and sonic virtual temperature measurements; (2) an Open CO_2_/H_2_O analyzer (LI-7500A, LI-COR Inc., Lincoln, NE, USA) for CO_2_ and H_2_O mixing ratio measurements; and (3) the open-path CH_4_ analyzer (LI-7700, LI-COR Inc., Lincoln, NE, USA) for CH_4_ molar density measurements.

Micrometeorological measurements for the variables in this study were also conducted near the EC system, especially for soil temperature (Ts, °C) and soil water content (SWC, m^3^/m^3^) at 0.05 m, 0.10 m, 0.20 m, 0.30 m and 0.50 m below the soil surface. The average of the five depth of data was used for analysis. LAI was measured weekly using an LAI-2200 Plant Canopy Analyzer (LI-COR, USA) under diffuse light conditions. Each value represents the average of three replicate readings from the center of the rice field.

Global and Asian flux-weighted CH_4_ emission source signature were obtained with annual EDGARv6.0 emission grid maps with the spatial resolution of 0.1 ° × 0.1 °.^68^ We calculated the average EDGAR CH_4_ emissions within the eight selected rice-producing countries constrained by latitude and longitude, respectively. Combined with the reported δ^13^C_source_ values of these eight countries (Table S2), the global and Asian flux-weighted δ^13^C_source_ were calculated.

### Quantification and statistical analysis

All statistical analyses were performed using Python 3.9 and OriginPro 2021. Pearson’s correlation coefficients were calculated to examine the relationships between δ^13^C_source_ and CO_2_ flux components (GPP and respiration) across the rice growing season. Regression analyses, including Keeling plot-based source signature estimation and model-based isotopic source partitioning, were implemented using custom Python scripts (see Data S1). Statistical significance was determined at *p* < 0.05 or *p* < 0.01, as specified. All results are reported as mean ± 1 standard deviation (1σ). Additional methodological and statistical details are provided in the figure legends and the method details section.

## Supplemental information

Document S1. Figures S1–S13 and Tables S1–S6

Data S1. Python code used for simulating isotopic signatures of methane emissions from plant-mediated and non-plant-mediated transport pathways, as well as for Keeling plot analyses, related to Figures 2 and 3

Data S2. Processed δ^13^CH_4_ and CH_4_ concentration data, together with CH4 flux, GPP, respiration, and relevant meteorological data from Jurong, related to Figures 2–4 and Supplementary Figures
