# Supplementary material for: Temporal variations of δ13C-CH4 in rice paddies dominated by the plant-mediated pathway
Source: iScience. 2025 Jun 16;28(7):112886. doi: 10.1016/j.isci.2025.112886 (PMC12266575; doi:10.1016/j.isci.2025.112886)
Supplement: Document S1. Figures S1–S13 and Tables S1–S6 [file mmc1.pdf]

**iScience, Volume *28***

## Supplemental information

# Temporal variations of $\delta^{13}C\text{-}CH_4$ in rice paddies dominated by the plant-mediated pathway

**Ji Li, Huilin Chen, Aijun Ding, Xuguang Chi, Weimin Ju, Yongguang Zhang, Philippe Ciais, Wenping Yuan, Shushi Peng, Zeqing Ma, Guirui Yu, and Jing M. Chen**

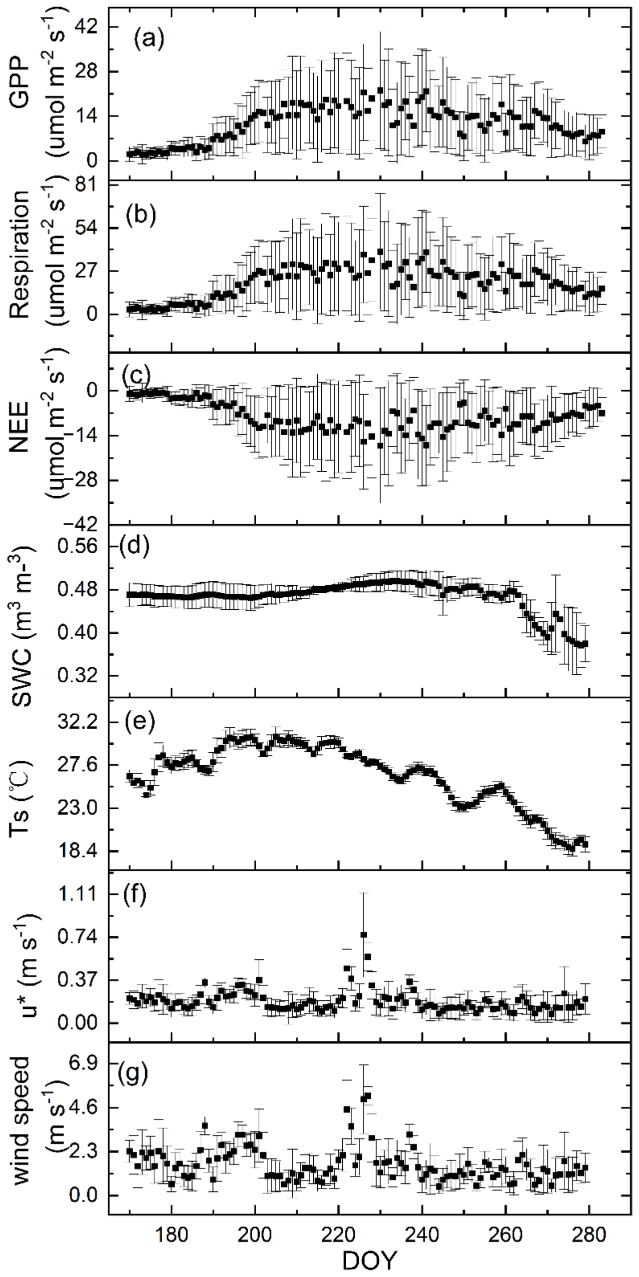


**Figure. S1. Seasonal dynamics of GPP, TER, NEE, and related meteorological variables during rice growth.**

Seasonality of daily (a) gross primary productivity (GPP), (b) Total ecosystem respiration, (c) net ecosystem exchange (NEE), (d) soil water content (SWC), (e) soil temperature (Ts), (f) surface friction velocity (u*) and (g) wind speed. The uncertainties are represented as 1-sigma standard deviation in error bars. Soil temperature and water content shown in this Figureure represent the average values across five depths (0.05 m, 0.10 m, 0.20 m, 0.30 m, and 0.50 m) below the surface. (Related to Figure. 2 and Figure. 4)

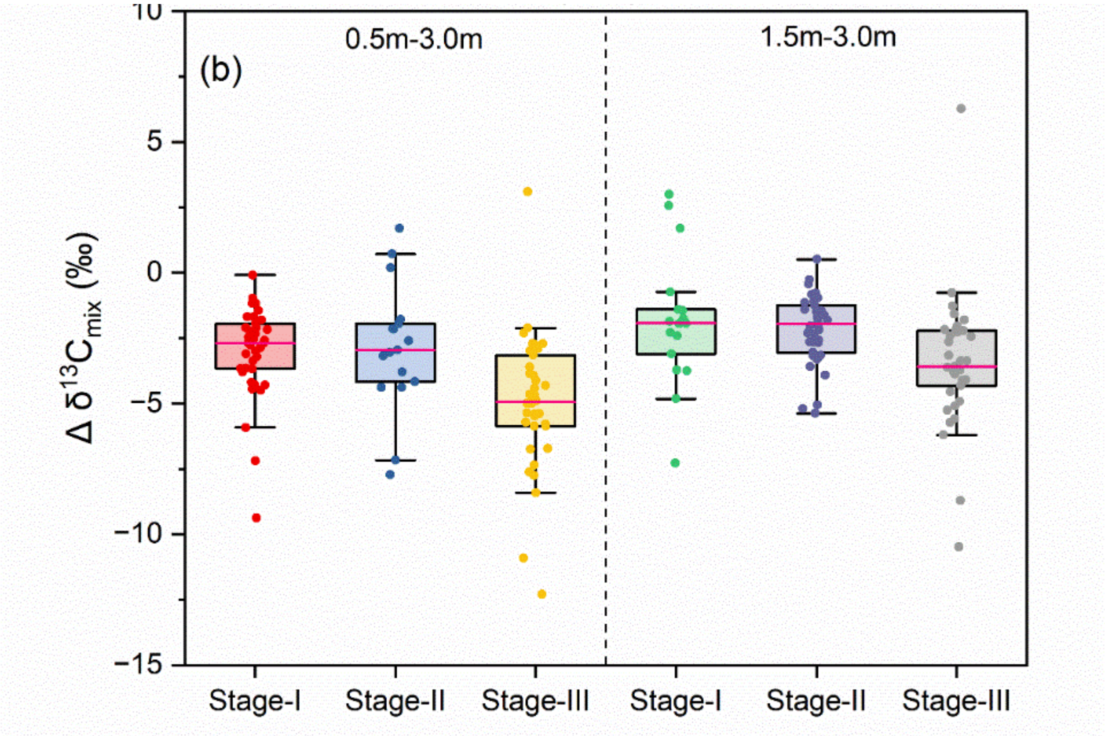


**Figure. S2. Vertical differences in daily $\delta^{13}CH_4$ source signatures estimated by Keeling plot.**

Difference of daily $\delta^{13}C$-$CH_4$ source signatures by Keeling plot at three different observation heights. $\delta^{13}C_{mix}$ derived from Keeling plot method based on daily group of 5-min $CH_4$ mole fraction and $\delta^{13}C$-$CH_4$ and was filtered by a threshold of $R^2 > 0.5$. $\delta^{13}Cmix$ on 0.5m, 1.5m and 3.0m are represented by green triangles, purple circles and black squares, respectively, to reflect the difference between three observation heights. (Related to Figure. 2)

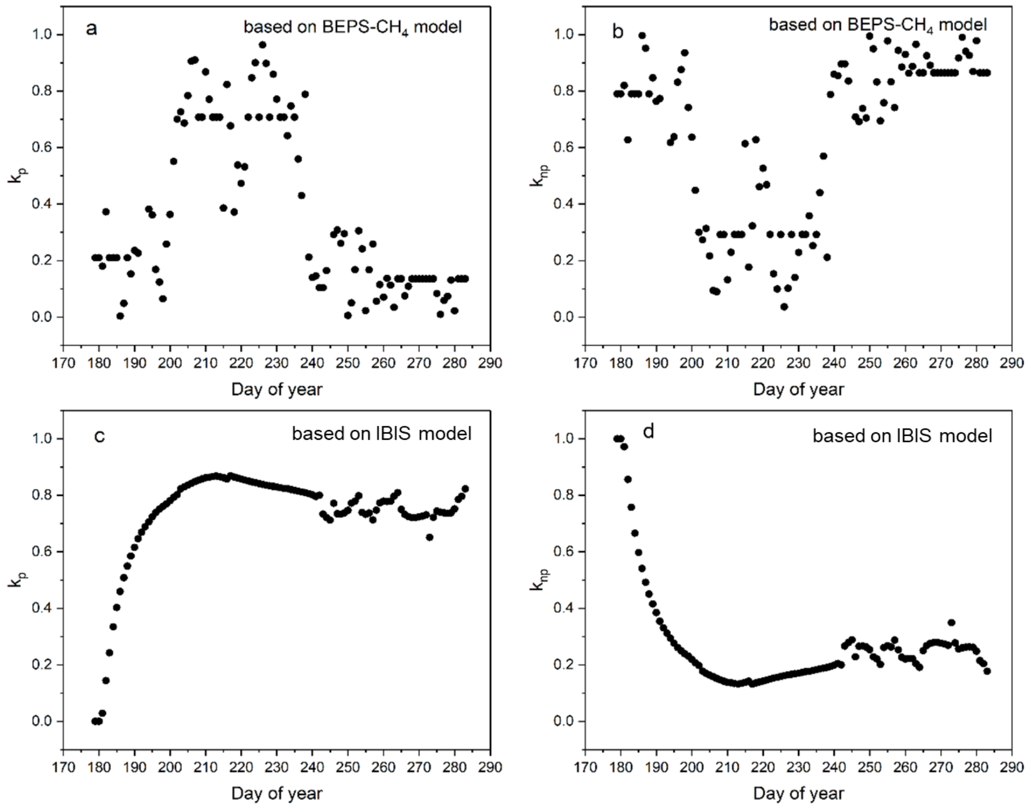


**Figure. S3. Seasonal variation of $k_p$ and $k_{np}$ derived from BEPS-$CH_4$ and IBIS models.**

Seasonality of $k_p$ and $k_{np}$ calculated from BEPS-$CH_4$ model and IBIS-model, respectively. By estimation the $CH_4$ flux emitted by plant-mediated and non-plant-mediated (included ebullition and diffusion) pathways from rice paddy, daily average $k_p$ and $k_{np}$ were calculated and applied for $\delta^{13}C_P$ and $\delta^{13}C_{NP}$ simulations. (Related to Figure. 3)

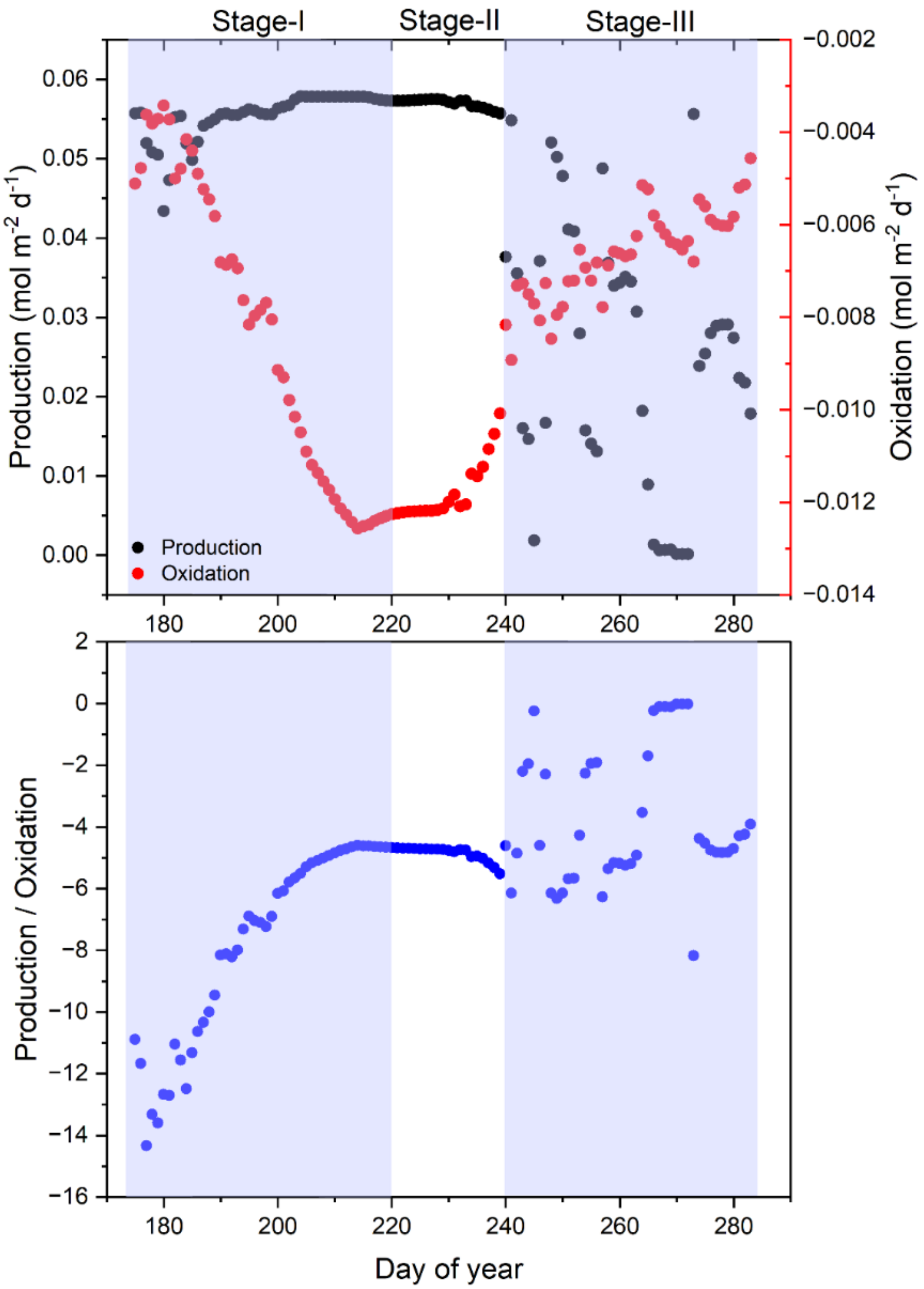


**Figure S4. Modelled $CH_4$ production, oxidation, and their ratio throughout the rice growing season.**

Seasonality of modelled $CH_4$ production, oxidation, and their ratio based on IBIS-model during rice growing season. (Related to Figure. 3)

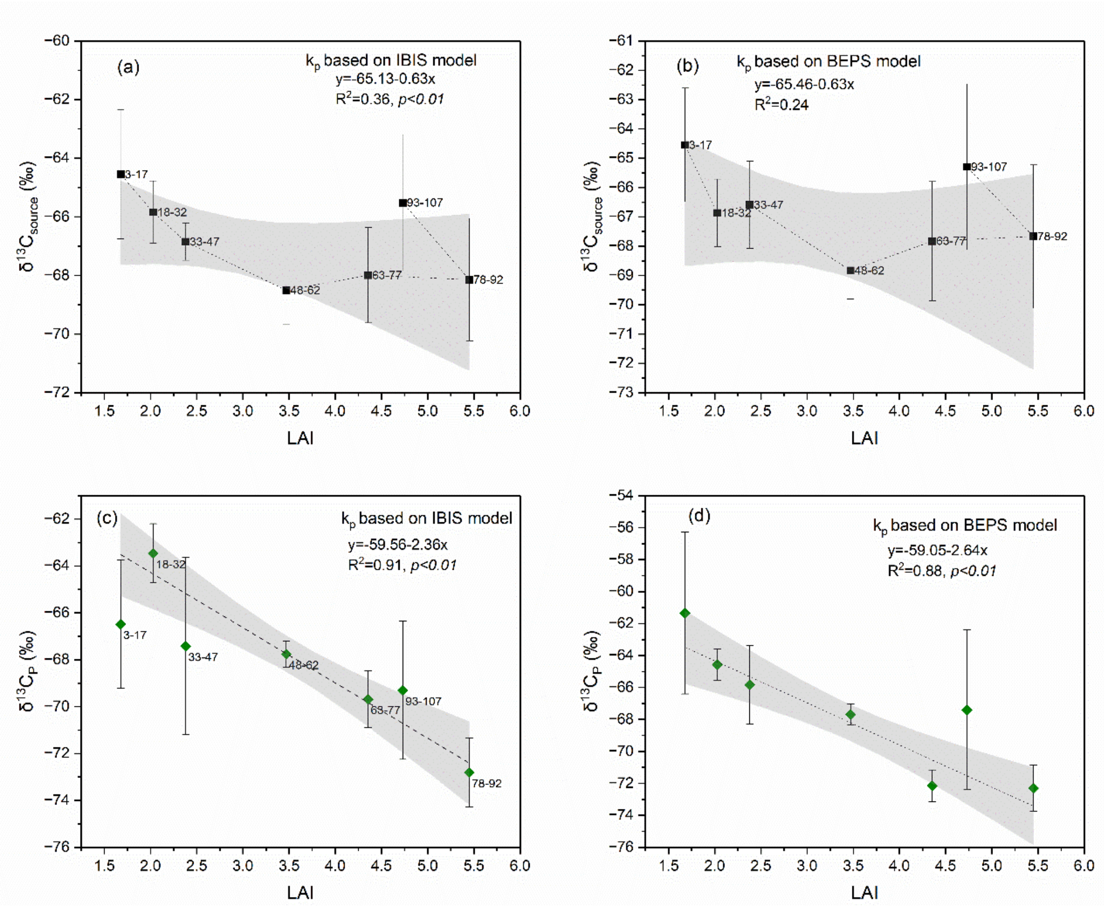


**Figure. S5. Correlations between LAI and $\delta^{13}C_{source}$ and $\delta^{13}C_P$ for 15-day groups.**

Seasonal correlations between LAI and $\delta^{13}C_{source}$ and $\delta^{13}C_P$ of every 15-day group. $\delta^{13}C_P$ was simulated based on $k_p$ were derived based on $k_p$, from (a) IBIS-model and (b) BEPS-$CH_4$ model, respectively. The numeric label represents the DOY of the group. The uncertainties are represented as 1-sigma standard deviation in error bars. (Related to Figure. 4)

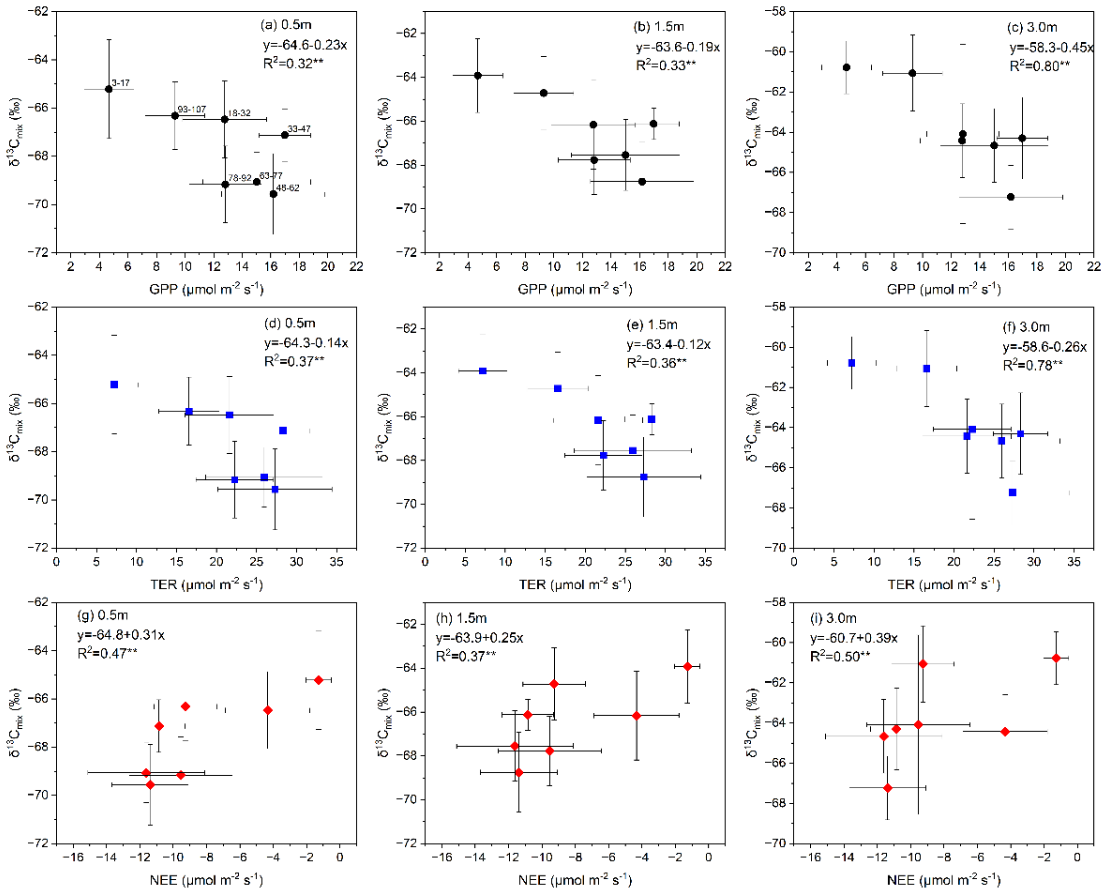

**Figure. S6. Correlations between carbon fluxes (GPP, TER, NEE) and $\delta^{13}C_{mix}$ at multiple heights.**

Seasonal correlations between (a, b, c) GPP, (d, e, f) TER, (g, h, i) NEE and $\delta^{13}C_{mix}$ at three different heights of every 15-day group. The hollow dots are the data of Stage-I and Stage-II. The numeric label represents the DOY of the group. The uncertainties are represented as 1-sigma standard deviation in error bars. (Related to Figure. 2 and Figure. 4)

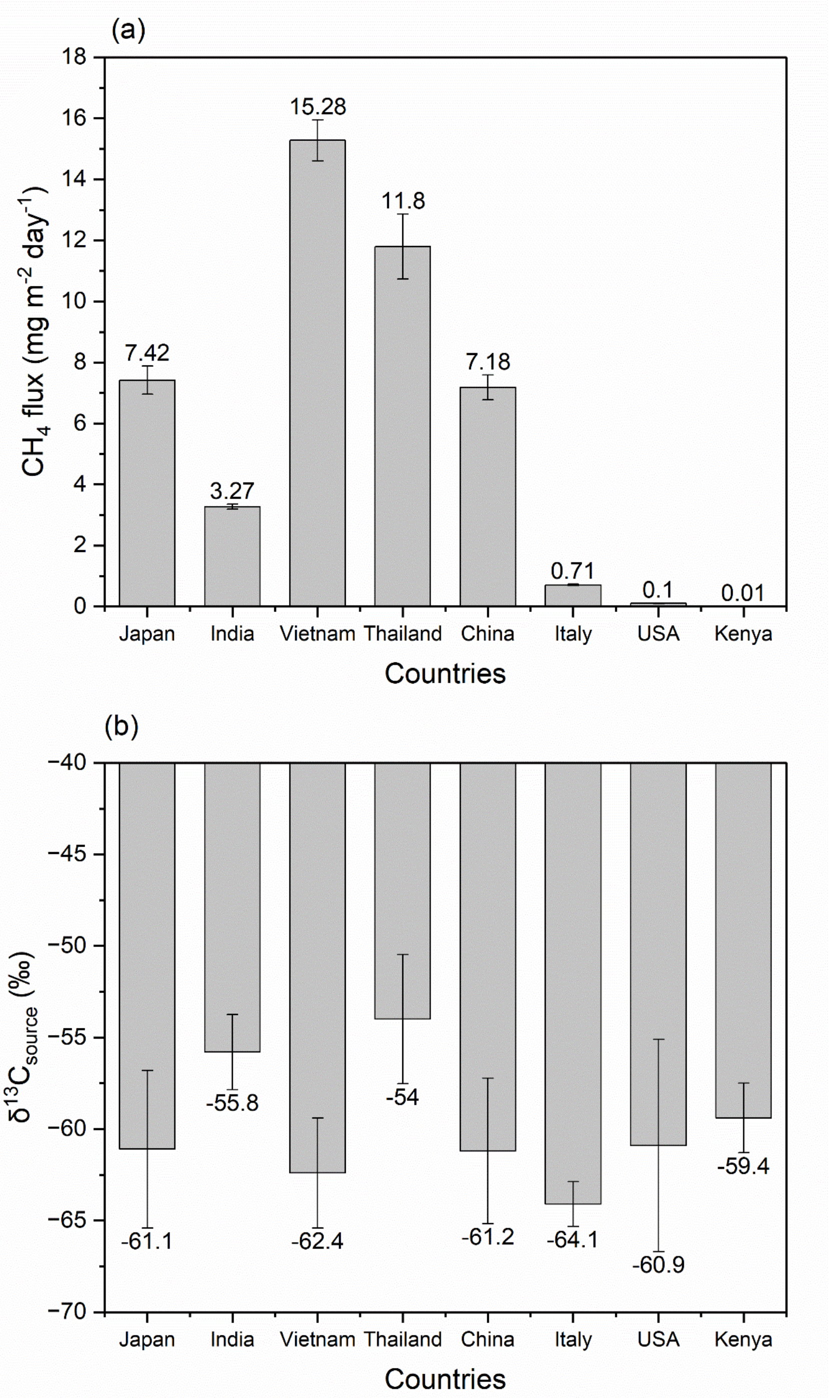


**Figure.S7. Inter-annual average $CH_4$ flux and $\delta^{13}C_{source}$ in major rice-producing countries.**

(a)The inter-annual average $CH_4$ flux and $\delta^{13}C_{source}$ for major rice-growing countries. The data comes from the EDGAR v6.0 greenhouse gases emission inventory and the reported studies. (Related to Figure. 4)

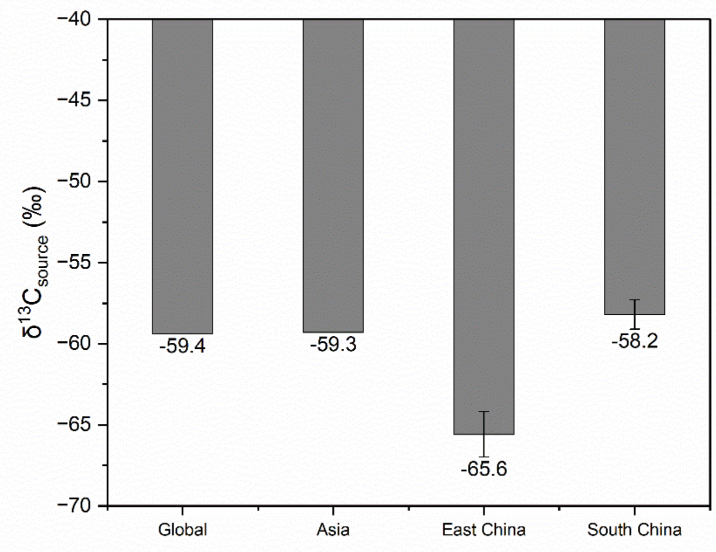


**Figure.S8. Distribution of $\delta^{13}C$-$CH_4$ for $CH_4$ emission sources.**

$\delta^{13}C_{source}$ values for global and Asian rice paddies were derived using a $CH_4$ flux-weighted method. Values for East China and South China were averaged from reported $\delta^{13}C_{source}$ values in Table S2. The uncertainties are represented as 1-sigma standard deviations. The "Global" category includes rice paddies in Europe (Italy), Asia (Japan, India, Vietnam, Thailand, China), North America (United States), and Africa (Kenya). $CH_4$ flux data for these locations were obtained from the EDGAR v6.0 inventory for the years 2000–2018 (interannual average $CH_4$ fluxes are shown in Figure S7). (Related to Figure 4)

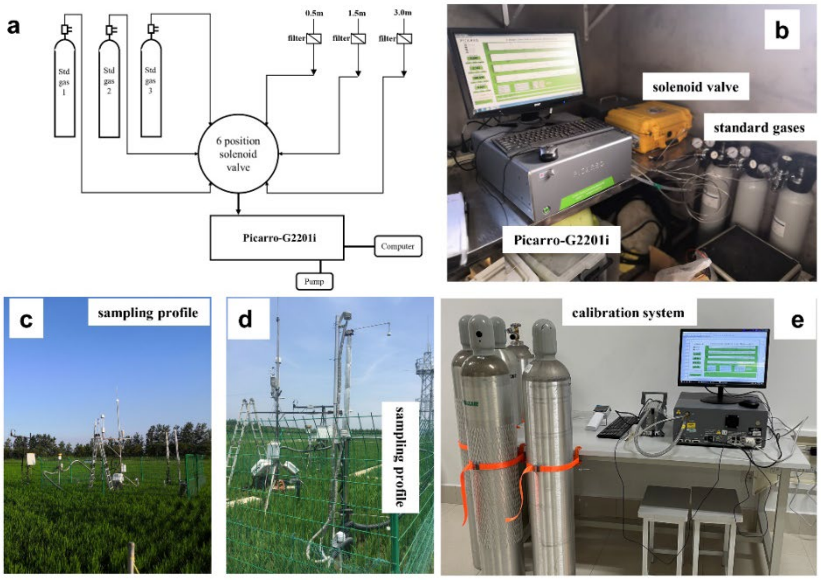


**Figure. S9. Field measurement and calibration setup systems.**

(a) Schematic of the set-up for field measurements based on Picarro-G2201i analyzer; (b-d) Photos of observation system and sampling profiles; (e) Photo of calibration system. (Related to STAR★METHODS)

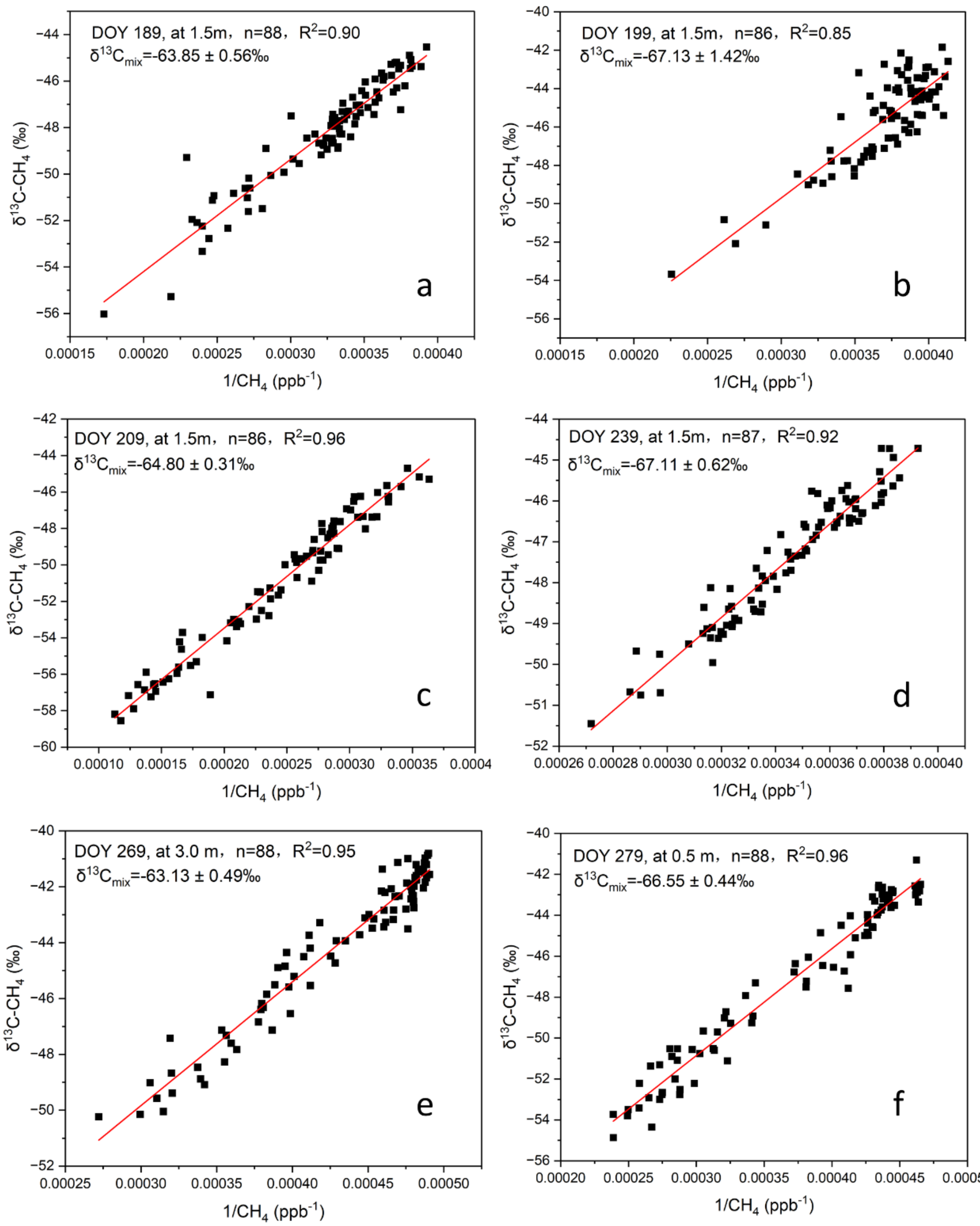


**Figure. S10. Examples of linear regression results for $\delta^{13}C_{mix}$ using Keeling plot method.**

Linear regression for deriving $\delta^{13}C_{mix}$ by Keeling plot method. Daily results in the Figureures were randomly selected to show the linear fitting results of Keeling plot. Significant differences and correlations were set at $p < 0.05$, and “n” represents the number of observations of every selected date. (Related to STAR★METHODS)

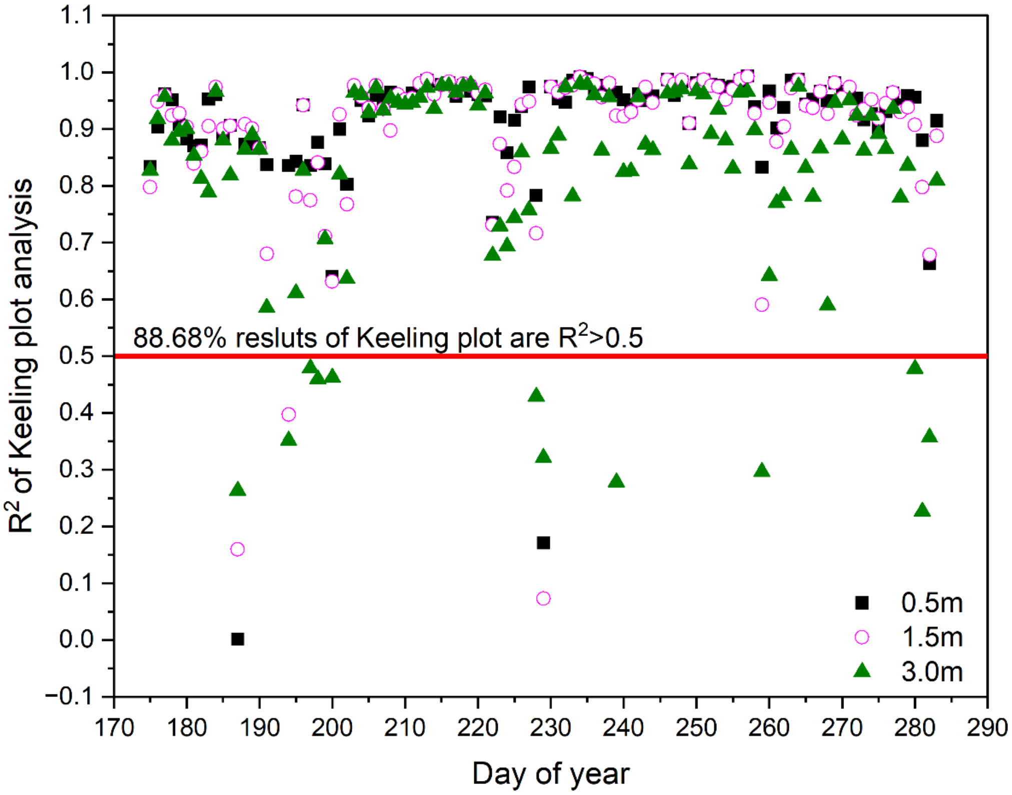


**Figure. S11. Summary of daily $\delta^{13}C_{mix}$ selection based on Keeling plot regression criteria.**

Data selection for daily $\delta^{13}C_{mix}$ derived by Keeling plot methods. Linear regression between observed $\delta^{13}C$-$CH_4$ and $1/CH_4$ were conducted and 88% of the results of $R^2 > 0.5$ shown in the Figureure above the red line were selected for the $CH_4$ source signature analysis. There are 106 results of $\delta^{13}C_{mix}$ from DOY175 to DOY 283 covering from the start of rice transplanting to the rice maturity. (Related to STAR★METHODS)

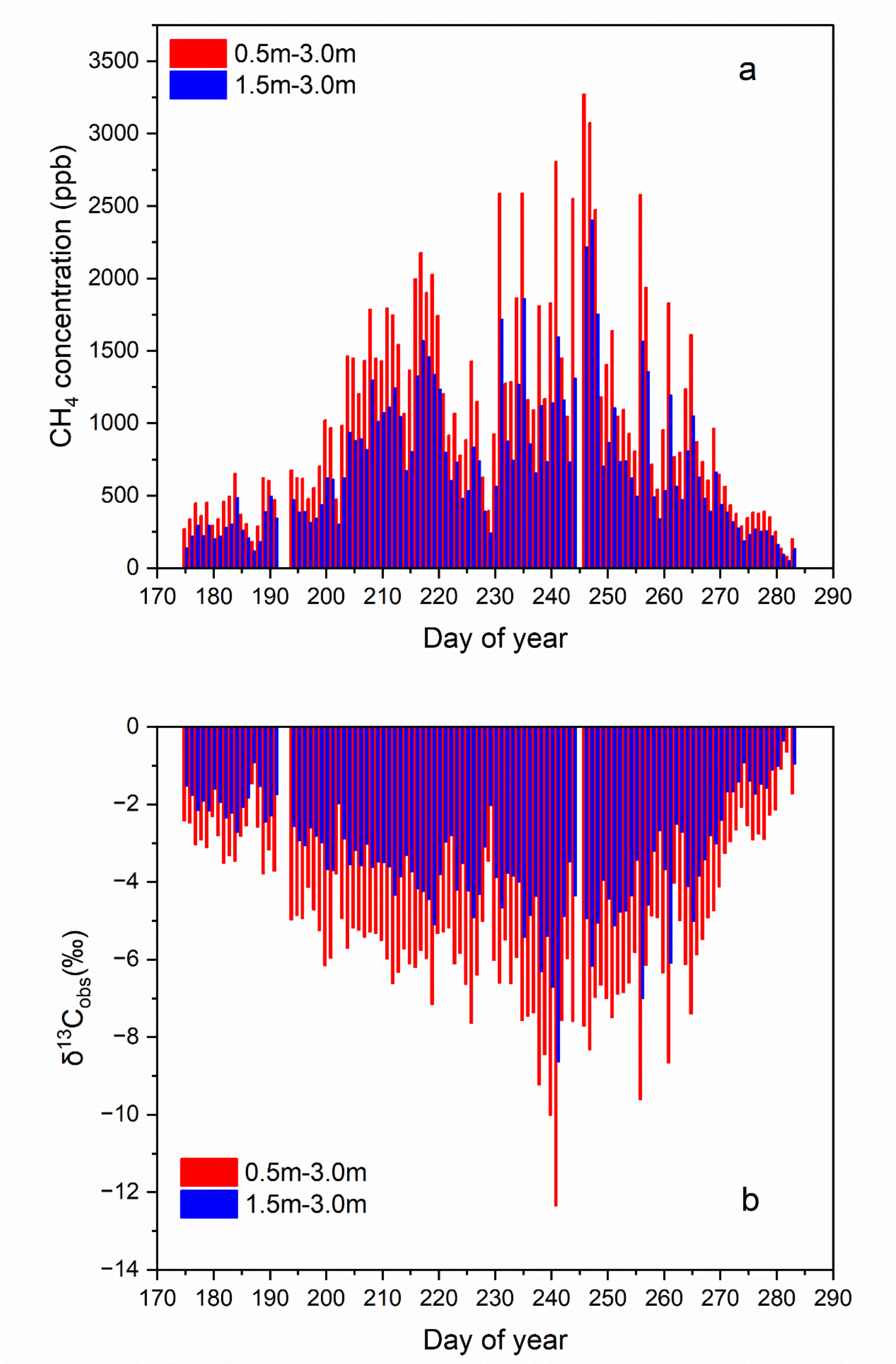


**Figure. S12. Vertical differences in $CH_4$ concentration and $\delta^{13}C_{obs}$ among observation heights.**

Difference of (a) $CH_4$ concentration and (b) $\delta^{13}C_{obs}$ between that at 0.5m and 3.0m, and between that at 1.5m and 3.0m, respectively. (Related to Figure. 2, Figure. 4 and STAR★METHODS)

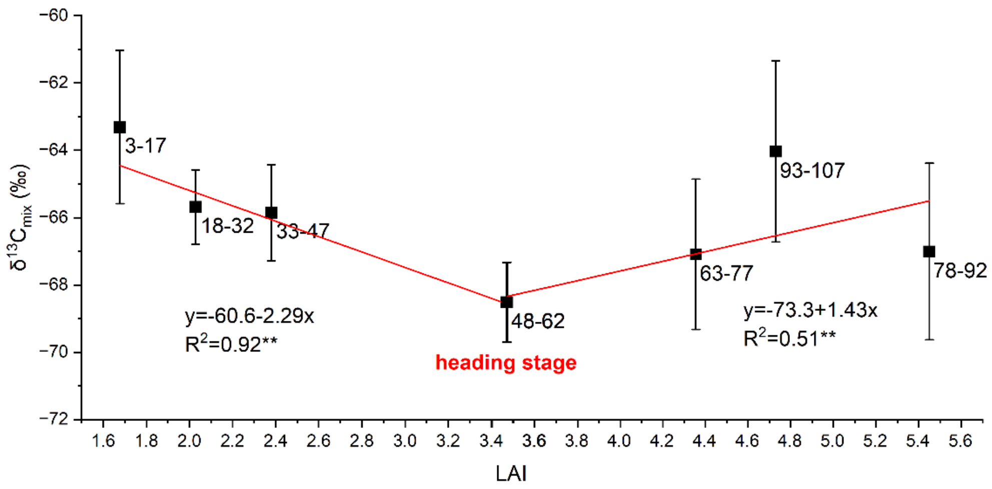


**Figure. S13. Correlations between LAI and $\delta^{13}C_{mix}$ averaged across different heights.**

Seasonal correlations between LAI and average $\delta^{13}C_{mix}$ from three different heights of every 15-day group. The numeric label represents the DOY of the group. The uncertainties are represented as 1-sigma standard deviation in error bars. (Related to Figure. 4)

**Table S1. Regression analysis between $CH_4$ flux and ecosystem/environmental variables in different stages.**

The regression between $CH_4$ flux and GPP, SWC, Ts in rice field. (Related to Figure. 2 and STAR★METHODS)

| | GPP (μmol/ $m^2$/ s) | TER (μmol/ $m^2$/ s) | SWC ($m^3$/ $m^3$) | Ts (°C) |
|---|---|---|---|---|
| **Stage-I** | 0.67** (L, +) | 0.66** (L, +) | - | 0.63** (L, +) |
| **Stage-II** | 0.10 (L, +) | 0.07 (L, +) | - | 0.74** (L, +) |
| **Stage-III** | 0.40** (EXP, +) | 0.38** (EXP, +) | 0.55** (EXP, +) | 0.88** (EXP, +) |

**represents $p<0.01$, *represents $p<0.05$. (+) represents the positive relationship and (-) represents the negative relationship. 'L' represents the linear regression, and 'EXP' represents the exponential fitting.

**Table S2. Isotopic source signatures of rice paddies from this study and previous publications.**

Summary of isotopic source signatures for rice paddies from this work and a review of previous published data. (Related to Figure. 4 and STAR★METHODS)

| Location | State region | $\delta^{13}C_{source}$ | error | type | Sampling information | reference |
|---|---|---|---|---|---|---|
| Italy | unspecified | -63.8 | 2.1 | 1 st. dev. | Across cultivation cycle | Krueger et al. 2002 |
| Italy | vercelli | -65.4 | 1.6 | 1 st. dev. | Single-point sampling | Levin et al. 1993 |
| Italy | vercelli | -63 | NA | NA | Flux weighted average throughout growing season; gas bubble collected with a funnel | Marik et al. 2002 |
| Japan | unspecified | -65.8 | 3.8 | 2 st. dev. | Rice 1990 and 1991 growing seasons | Tyler et al. 1994 |
| Japan | unspecified | -63.1 | 4.9 | 1 st. dev. | Rice 1990 and 1991 growing seasons | Tyler et al. 1994 |
| Japan | unspecified | -55.9 | 4.2 | 1 st. dev. | Ten different locations throughout country | Uzaki et al. 1991 |
| Japan | yokohama | -59.6 | 3.4 | 1 st. dev. | Seasonal variation in Yokohama | Uzaki et al. 1991 |
| Kenya | unspecified | -59.4 | 1.9 | 1 st. dev. | NA | Tyler et al. 1988 |
| USA | california | -67.2 | 1 | 1 st. dev. | Aug | Stevens and Engelkemeir 1988 |
| USA | louisiana | -64.5 | 1 | 1/2 of range | NA | Chanton et al. 1997 |
| USA | louisiana | -63.2 | 2.9 | 1 st. dev. | NA | Wahlen et al. 1989 and 1990 |
| USA | texas | -56.1 | 1.2 | 1 st. dev. | Flux weighted average throughout growing season | Tyler et al. 1997 |
| USA | texas | -53.6 | 1 | 1 st. dev. | Flux weighted average throughout growing season | Tyler et al. 1997 |
| South China | Hong Kong | -58.7 | 0.4 | 1 st. dev. (keeling plot) | NA | Brownlow et al. 2017 |
| South China | Hong Kong | -58.9 | 0.4 | 1 st. dev. | NA | Brownlow et al. |

| | | | | | | |
|---|---|---|---|---|---|---|
| | | | | (keeling plot) | | 2017 |
| South China | Hong Kong | -57 | 0.3 | 2 st. dev. | Yi O Rice 2016–2019 combined dataset | France et al. 2022 |
| South China | Hong Kong | -59.1 | 0.8 | 1 st. dev. | Yi O Rice 2017 growing season | France et al. 2022 |
| South China | Hong Kong | -57.2 | 0.4 | 1 st. dev. | Yi O Rice 2018 growing season | France et al. 2022 |
| South China | Hong Kong | -58.2 | 1.7 | 1 st. dev. | Yi O Rice 2019 growing season | France et al. 2022 |
| India | andhra pradesh | -54.3 | NA | NA | Flux weighted average throughout growing season | Rao et al. 2008 |
| India | gujarat | -57.2 | NA | NA | Flux weighted average throughout growing season | Rao et al. 2008 |
| Vietnam | Ho Chi Min | -62.4 | 3 | 1 st. dev. | Ho Chi Min City post-harvest | France et al. 2022 |
| Thailand | Southern Thailand | -56.5 | 4.6 | 1 st. dev. | Across cultivation cycle; gas bubbles stirred up with rod; peat soils | Nakagawa et al. 2002 |
| Thailand | Southern Thailand | -51.5 | 7.1 | 1 st. dev. | Across cultivation cycle; gas bubbles stirred up with rod; mineral soils | Nakagawa et al. 2002 |
| East China | Suzhou | -63.8 | 4.9 | 1 st. dev. | Across cultivation cycle | Bergamaschi et al. 1997 |
| East China | Zhenjiang | -65.1 | 3.6 | 1 st. dev. | Jurong Rice 2009 growing season; flux chambers | Zhang et al.2016 |
| East China | Zhenjiang | -64.6 | 7.2 | 1 st. dev. (keeling plot) | in-situ continuous observation during rice growing season; by keeling plot method | This study |
| East China | Zhenjiang | -66.8 | 1.5 | 1 st. dev. | in-situ continuous observation during rice growing season; weighted average values based on $\delta^{13}C_P$ and $\delta^{13}C_{NP}$ | This study |
| East China | Zhenjiang | -66.7 | NA | NA | Flux weighted average throughout growing season | This study |

**Table S3. Regression analysis of $CH_4$ flux, friction velocity, and wind speed during rice growth.**

The linear regression between $CH_4$ flux, u* and wind speed and $CH_4$ mole fraction in rice growth stages. (Related to Figure. 2 and STAR★METHODS)

| | $CH_4$ flux (μmol/ $m^2$/ s) | | | u* (m/s) | | | wind speed (m/s) | | |
|---|---|---|---|---|---|---|---|---|---|
| | 0.5m | 1.5m | 3.0m | 0.5m | 1.5m | 3.0m | 0.5m | 1.5m | 3.0m |
| **Stage-I** | 0.41** (+) | 0.38** (+) | 0.24* (+) | 0.30** (-) | 0.33** (-) | 0.39** (-) | 0.34** (-) | 0.38** (-) | 0.44** (-) |
| **Stage-II** | - | - | - | 0.51** (-) | 0.50** (-) | 0.48** (-) | 0.56** (-) | 0.55** (-) | 0.53** (-) |
| **Stage-III** | 0.44** (+) | 0.39** (+) | 0.23* (+) | 0.15* (-) | 0.18* (-) | 0.24** (-) | 0.13 (-) | 0.16 (-) | 0.22** (-) |

**represents $p<0.01$, *represents $p<0.05$. (+) represents the positive relationship and (-) represents the negative relationship. '-' represents that there was no significant correlation in this growth stage.

**Table S4. Classification of rice growth stages and associated $CH_4$ emission pathways.**

Rice growth stages and $CH_4$ emission pathways. (Related to STAR★METHODS)

| Classification in this study | Transition sign | Category | Day of year | Days after transplanting | Growth stage | Water management | Fertilizer application |
|---|---|---|---|---|---|---|---|
| ~ | Germination | ~ | 165~176 | ~ | Seeding | Start to flood | Based fertilizer |
| | | | | | Transplanting | | |
| **Stage-I** | | Vegetative | 176-220 | 0-44 | Tillering | Continued flooding | Tillering fertilizer |
| | Panicle differentiation | | | | Jointing | | |
| **Stage-II** | Heading | Vegetative & Reproductive | 221-240 | 45-64 | Booting Heading | Continued flooding | Panicle fertilizer |
| **Stage-III** | | | 241-250 | 65-75 | Flowering | Saturated but not flooded | |
| | | Reproductive | 251-270 | 76-95 | Milk filling | Drainage | |
| | Maturation | | 271-283 | 96-107 | Repining | | |

**Table S5. Reference $CH_4$ concentrations and $\delta^{13}CH_4$ values for standard gases.**

Standard gas composition used for $\delta^{13}CH_4$ calibration, including $CH_4$ mixing ratios, isotopic signatures, and calculated $^{12}CH_4$ and $^{13}CH_4$ mole fractions. (Related to STAR★METHODS)

| Reference Standard | $CH_4$ (ppb) | $\delta^{13}CH_4$ (‰) | $^{13}r$ | $R_{sum}$ | $^{13}CH_4$ (ppb) | $^{12}CH_4$ (ppb) |
|---|---|---|---|---|---|---|
| | | | | | True values | |
| Std1 | 2004.32 | -46.80 | 0.010656929 | 1.011223724 | 1982.07 | 21.12 |
| Std2 | 3592.80 | -47.01 | 0.010654656 | 1.011221449 | 3552.93 | 37.86 |
| Std3 | 5017.03 | -47.16 | 0.010652979 | 1.011219770 | 4961.36 | 52.85 |

**Table S6. Vertical gradient of $CH_4$ concentrations and $\delta^{13}C_{obs}$ at different observation heights.**

Vertical Gradient of $CH_4$ mole fractions and $\delta^{13}C_{obs}$ among three different observation heights. (Related to Figure. 2 and STAR★METHODS)

| Vertical Gradient | $CH_4$ mole fractions | $\delta^{13}C_{obs}$ |
|---|---|---|
| 0.5m-3.0m | 80.7ppb ~ 3740.4ppb | -12.4‰~-0.7‰ |
| 1.5m-3.0m | 50.0ppb ~ 2403.2ppb | -8.6‰~-0.0‰ |
